# Optimization of formulation for enhanced intranasal delivery of insulin with translationally controlled tumor protein-derived protein transduction domain

**DOI:** 10.1080/10717544.2019.1628119

**Published:** 2019-06-18

**Authors:** Hae-Duck Bae, Ji-Sun Lee, Haejun Pyun, Moonhee Kim, Kyunglim Lee

**Affiliations:** Graduate School of Pharmaceutical Sciences, College of Pharmacy, Ewha Woman’s University, Seoul, Korea

**Keywords:** Drug formulation, insulin, intranasal delivery, protein transduction domain, translationally controlled tumor protein

## Abstract

Intranasal delivery of insulin is an alternative approach to treat diabetes, as it enables higher patient compliance than conventional therapy with subcutaneously injected insulin. However, the use of intranasal delivery of insulin is limited for insulin’s hydrophilicity and vulnerability to enzymatic degradation. This limitation makes optimization of formulation intranasal insulin for commercial purpose indispensable. This study evaluated bioavailability (BA) of various formulations of insulin intranasally delivered with protein transduction domain (PTD) derived from translationally controlled tumor protein. The therapeutic efficacy of newly formulated intranasal insulin + PTD was compared *in vivo* studies with normal and alloxan-induced diabetic rats, to those of free insulin and subcutaneously injected insulin. BA of insulin in two new formulations was, respectively, 60.71% and 45.81% of subcutaneously injected insulin, while the BA of free insulin was only 3.34%. Histological analysis of tissues, lactate dehydrogenase activity in nasal fluid, and biochemical analysis of sera revealed no detectable topical or systemic toxicity in rats and mice. Furthermore, stability analysis of newly formulated insulin + PTD to determine the optimal conditions for storage revealed that when stored at 4 °C, the delivery capacity of insulin was maintained up to 7 d. These results suggest that the new formulations of intranasal insulin are suitable for use in diabetes therapy and are easier to administer.

## Introduction

Intranasal administration is a non-invasive route which has been applied to effective delivery of broad range of drugs including small molecule drugs and macromolecular drugs (Hussain, 1998; Fortuna et al., 2014; Avgerinos et al., 2018). A number of small molecule drugs (e.g. butorphanol, estradiol, naloxone, ands sumatriptan) have already been marketed as nasal formulations (Mathias & Hussain, 2010). Nasal small molecule drugs can reach general circulation a few minutes after the administration, thus, being effective when needed urgently. Nasal peptide- or protein-based drugs (e.g. desmopressin, oxytocin, and nafarelin) have also been marketed or are under development. Most parenteral drugs for biomacromolecule are extremely susceptible to enzymatic degradation in gastrointestinal tract. Thus, the main administration route remains invasive subcutaneous injection. However, recent studies attempting to find various formulations for effective delivery of those drugs focused on nasal administration as an alternative route for biomacromolecules. Advantages of intranasal administration center on the anatomical features of nasal mucosa. When drugs are administrated by intranasal route, they mainly entered through respiratory region around the inferior turbinate. Respiratory nasal mucosa is highly permeable and vascularized and is lined with columnar epithelium consisted of various cell types which confer large surface area for systemic drug absorption (Pires et al., 2009; Grassin-Delyle et al., 2012). The intranasal route avoids proteolytic digestion that occurs in the gastrointestinal tract and first-pass hepatic metabolism, thus, avoiding problems which limit drug absorption. Among various drugs which are under the development for intranasal administration, insulin ranks high (el-Etr et al., 1987; Frauman et al., 1987b; Chandler et al., 1994; Dyer et al., 2002; D'Souza et al., 2005). Current insulin formulation for subcutaneous injection has the weakness of the patient compliance because of the inconvenience of the injection, which frequently leads to non-compliance. Both increased patient compliance and exhibiting pharmacokinetics identical with that of subcutaneously delivered insulin (Frauman et al., 1987a) make this route of administration very attractive in the treatment of diabetes. However, there remain several issues that should be addressed before accepting the suitability of intranasal delivery of insulin in diabetes therapy. These include the need for absorption enhancers and for increasing the residence time of the drug in the nasal cavity (Hinchcliffe & Illum, 1999; Arora et al., 2002; Owens et al., 2003). Thus, the use of intranasal insulin still awaits development of optimized formulations that overcome the noted limitations.

Protein transduction domains (PTDs), also known as cell penetrating peptides, are short peptides that transduce cellular membranes without intervention of specific receptors (Vives et al., 1997; Schwarze et al., 2000; Jarver & Langel 2004; Gupta et al., 2005). PTDs translocate into cellular compartment without damaging cellular membrane, which makes them promising carriers to deliver macromolecules. It has been reported that broad ranges of molecules, such as DNA, small interfering RNA, proteins, and nanoparticles, translocate into cells mediated by PTDs (Guidotti et al., 2017). Furthermore, delivered molecules maintain their biological effects both in *in vitro* and *in vivo* (Morris et al., 2001; Meade & Dowdy 2007; Kim et al., 2011b; Bae et al., 2016). Recently, hydrophilic peptide drugs which are limited to intranasal administration were effectively delivered into systemic circulation without damage of nasal mucosal membrane (Choi et al., 2006; Khafagy et al., 2009; Sakuma et al., 2010). Especially effective intranasal delivery of insulin was reported to have been achieved by mixing with PTDs (Khafagy et al., 2013). This study showed that L-forms of octagonarginine, significantly increased delivery of insulin across the nasal membrane, pointing to the potential of PTDs as absorption promoters in the development of intranasal insulin.

We previously reported that the 10 amino acid sequence (MIIYRDKLISH) at the N-terminal of human translationally controlled tumor protein (TCTP) acts as a PTD (Kim et al., 2011b). Further studies have been conducted to develop or identify other peptides derived from TCTP-PTD with improved transduction activity (Kim et al., 2011a; Bae & Lee, 2013; Bae et al., 2018). Thus, we identified L-TCTP-PTD 13 (MIIFRALISHKK) and L-TCTP-PTD 13M2 (MIFRLLASHKK) as modified TCTP-PTDs with enhanced delivery capability for insulin when administered by nasal route. Although we confirmed therapeutic effectiveness of the intranasally delivered insulin with PTD, there remained several needs including sustained drug effect and improving storage conditions that avoid protein aggregation. In this study, we tried to address these needs and design and optimize TCTP-PTD-based formulations for nasal delivery of insulin.

## Materials and methods

### Materials and animals

Modified TCTP-PTD with N-terminal acetylation and C-terminal amidation was synthesized by Peptron Co., Ltd. (Daejeon, Korea). All other chemicals were purchased from Sigma-Aldrich (St. Louis, MO). Male Wistar rats and ICR mice (5-week old) were purchased from Young Bio Co., Ltd. (Seoungnam, Korea). They were housed under a 12 h-h light/dark conditions, controlled humidity and temperature with free access to food and water. For intranasal administration, animals were anesthetized by intraperitoneal injection of sodium pentobarbital and formulated insulin + PTDs were administered into the right nostril of the mice, using a pipette. All animal experiments were approved by Ewha Womans University’s Institutional Animal Care and Use Committee.

### Measurement of blood glucose in rats

Male Wistar rats were fasted overnight with free access to water. Those with fasting glucose level range of 90–120 mg/dL were selected for further experiments. Formulated insulin + PTDs were intranasally administered to the anesthetized rats. Blood samples were collected from the rat tails and their glucose levels were measured using an Accu-Chek glucose meter (Roche Diagnostics, Seoul, Korea). For generating the rat model of diabetes, alloxan (100 mg/kg, dissolved in 10 mM sodium citrate buffer, pH 3.2) was intraperitoneally injected into normal rats. After 5 d, rats with fasting blood glucose level range of 230–300 mg/dL were fasted overnight with free access to water, then anesthetized followed by intranasal administration. Blood glucose levels were measured in the same method.

### Lactate dehydrogenase (LDH) activity analysis

Fifteen minutes after intranasal administration of each insulin formulation or 5% sodium taurodeoxycholate (NaTDC), the nasal cavities were flushed out with 1 mL PBS. LDH activity in the washings was measured using a CytoTox 96 assay kit (Promega, Madison, WI) according to the manufacturer’s protocol.

### Plasma insulin measurement

After intranasal administrations, bloods were collected from the rat tails at desired time points and centrifuged at 5000 *g* for 25 min to obtain plasmas. The plasma insulin concentrations were measured by enzyme-linked immunosorbent assay (ELISA, Mercodia, Uppsala, Sweden) according to the manufacturer’s protocol.

### Toxicity test using mice

Male ICR mice were intranasally administered with insulin + PTD formulation once a day for 10 d. LDH activity was measured as described previously. The collected bloods were centrifuged for 25 min at 5000 *g* to obtain the plasma. Blood urea nitrogen (BUN), creatinine (CRE), aspartate aminotransferase (AST), and alanine aminotransferase (ALT) levels were determined using a biochemical analyzer (AU680; Beckman Coulter, Tokyo, Japan). After sacrifice, tissues were dissected and fixed in 4% formaldehyde overnight. Paraffin-embedded tissues were sectioned with microtome then stained with hematoxylin and eosin (H&E) solution and histological analysis was performed under the microscope.

### Statistical analysis

Data were analyzed using Prism 5 software (GraphPad Inc., La Jolla, CA) and presented as mean ± standard deviation (s.d.). *p* < .05 by Student’s *t*-test or one-way analysis of variance (ANOVA) followed by the Newman–Keuls multiple comparison test was considered statistically significant.

## Results and discussion

### Selection of formulation for intranasal delivery of insulin with PTD

Modified peptides derived from TCTP (L-TCTP-PTD 13, MIIFRALISHKK) have been reported to be an effective carriers for intranasal delivery of insulin. Double modification of residues at positions 6 and 8 of TCTP-PTD 13 (L-TCTP-PTD 13M2, MIIFRLLASHKK) improved intranasal delivery without toxicity in mice (Bae & Lee, 2013; Bae et al., 2018). In addition, optimized formulation for TCTP-PTD-based nasal delivery of insulin has been identified and this formulation which used arginine hydrochloride (ArgHCl) as an aggregation suppressor and sucrose as an osmolyte showed improved bioavailability (BA) and significant blood glucose-lowering effects in mice (Kim et al., 2019). However, ArgHCl has been reported to exert dual effects on protein aggregation (Smirnova et al., 2015; Borzova et al., 2017), thus further design for optimization considering concentration of sucrose and PTD with various pH was conducted (Table 1).

**Table 1.** Summary of intranasal insulin + PTD formulations.

Formulation	Insulin (mM)	TCTP-PTD 13M2 (mM)	Diluent	Aggregation suppressor	Surfactant	Antioxidant	Buffer
3–1 formulation	0.1	0.2	1 % sucrose	100 mM Arginine	Poloxamer 188 (0.5 mg/mL)	1 mM methionine	10 mM PB, pH 6.4
3–2 formulation	0.1	0.2	1 % sucrose	–	Poloxamer 188 (0.5 mg/mL)	1 mM methionine	10 mM PB, pH 6.4
3–3 formulation	0.1	0.25	1 % sucrose	–	Poloxamer 188 (0.1 mg/mL)	1 mM methionine	10 mM PB, pH 6.4
3–4 formulation	0.1	0.3	1 % sucrose	–	Poloxamer 188 (0.1 mg/mL)	1 mM methionine	10 mM PB, pH 6.4
3–5 formulation	0.1	0.25	1 % sucrose	–	Poloxamer 188 (0.1 mg/mL)	1 mM methionine	10 mM PB, pH 7
3–6 formulation	0.1	0.2	0.5 % sucrose	–	Poloxamer 188 (0.5 mg/mL)	1 mM methionine	10 mM PB, pH 6.4
3–7 formulation	0.1	0.2	0.25 % sucrose	–	Poloxamer 188 (0.5 mg/mL)	1 mM methionine	10 mM PB, pH 6.4
3–8 formulation	0.1	0.2	0.5 % sucrose	–	Poloxamer 188 (0.5 mg/mL)	1 mM methionine	10 mM PB, pH 7

**Figure 1.**
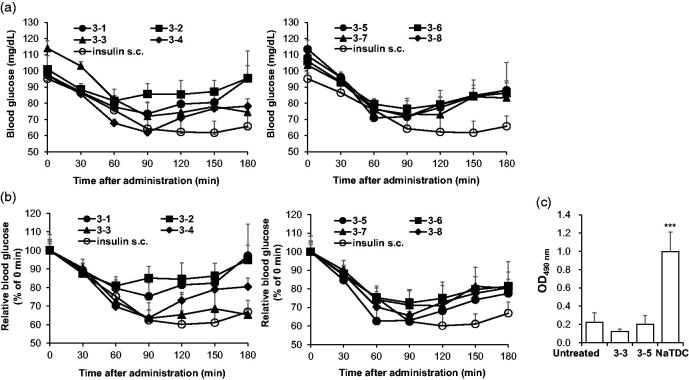
Selection of effective formulations for intranasal insulin + PTD with low toxicity. (a–c) Overnight-fasted normal rats were intranasally administered with each formulations the blood glucose levels were determined. The insulin dose was 5 IU/kg and 0.25 IU/kg for nasal route and s.c. injection, respectively (*n* = 4–7). (a) Blood glucose in normal rats after intranasal administration. Data from insulin s.c. of both graphs originated from the same experiment. (b) Relative blood glucose level relative to 0 min was calculated as shown as graph. As in (a), data from insulin s.c. of both graphs originated from the same experiment. (c) OD_490nm_ means LDH activity from nasal wash solution. 5% NaTDC was used as positive control known to be toxic. ***p < .001 by Student’s *t*-test compared to untreated group. Data are presented mean ± s.d.

To evaluate the pharmacodynamics of each formulation, blood glucose levels in normal rats after intranasal administration was monitored within 180 min (Figure 1(a)). For comparison of blood glucose lowering effect of each formulation, blood glucose at 0 min were set to 100% then relative blood glucose levels were calculated (Figure 1(b)). Among eight formulations, 3–3 and 3–5 were the two formulations which showed blood glucose-lowering effects in rats. Both formulations exerted up to 60% reduction which is comparable to efficacy of subcutaneously injected insulin, the conventional route for insulin therapy. To determine whether these formulations induce nasal membrane damage, LDH activity in the nasal fluid (Figure 1(c)) was measured. This intracellular enzyme activity has been used as an indicator of leakage of cytosolic constituent (Shao et al., 1992). While 5% NaTDC, positive control for nasal membrane damage, showed significantly increased LDH activity, 3–3 and 3–5 formulations did not show any detectable increase in LDH activity, indicating no toxicity from both formulations. Thus, we selected two formulations (3–3 and 3–5) which showed comparable efficacy to subcutaneously injected insulin with marginal toxicity for intranasal delivery of insulin and performed further analysis

### *In vivo* BA of insulin in formulated insulin + PTDs

**Figure 2.**
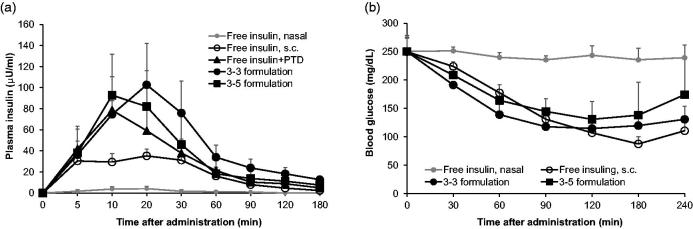
Pharmacokinetic and pharmacodynamic analysis of intranasal insulin + PTD formulations. (a) Normal rats were administered as indicated, followed by plasma insulin measurement. Insulin dose was 5 IU/kg for nasal route, 0.25 IU/kg for s.c. injection, and 1 IU/kg for administration with PTD (*n* = 5–8). (b) Blood glucose in alloxan-induced diabetic rats following each administration. Insulin dose was 2 IU/kg for nasal route, 1 IU/kg for s.c. injection, and 2 IU/kg for administration with PTD (*n* = 5–7). Data are presented mean ± s.d.

To evaluate whether the formulations improve intranasal delivery of insulin, we measured plasma insulin levels after intranasal administration in normal rats (Figure 2(a) and Table 2). When insulin (5 IU/kg) alone was administered intranasally, insulin was hardly detectable in plasma but when mediated by PTD, plasma insulin markedly increased in normal rats even in low insulin dose (1 IU/kg). Furthermore, both 3–3 and 3–5 formulations more effectively delivered insulin through the nasal epithelium when compared to free insulin + PTD. The insulin BA in each group was calculated relative to subcutaneously injected free insulin (taken as 100%). BA of insulin from 3–3 and 3–5 formulations was 60.71 ± 7.48% and 45.96 ± 6.12%, respectively, while the value of free insulin + PTD without formulation was 38.66 ± 4.51%. Next, we analyzed hypoglycemic effect of each formulation in the rat model of alloxan-induced diabetes to confirm the therapeutic effect of formulated insulin + PTD (Figure 2(b) and Table 3). The dose of insulin (2 IU/kg) of intranasally administered was based on our previous study that studied measurable hypoglycemic effect (Bae et al., 2018). Intranasally administered insulin showed likewise no blood glucose lowering effects, but formulated insulin + PTD remarkably lowered blood glucose after intranasal administration. Hypoglycemic effect of each formulation was maintained for 240 min. Pharmacological availability (PA) value in each group was calculated relative to subcutaneously injected free insulin (presented as 100%). PA values of 3–3 and 3–5 formulations were 49.33 ± 2.71% and 37.52 ± 5.64%, respectively, while the value of free insulin + PTD was only 4.01 ± 1.99%. Collectively, these results indicate that 3–3 and 3–5 formulations improve intranasal delivery and therapeutic efficacy of insulin + PTD.

**Table 2.** Pharmacokinetics of nasally administered insulin formulations.

Formulation	Insulin dose (IU/kg)	Route of administration	AUC_0–180 min_	Bioavailability (%)
Free insulin, nasal	5 IU/kg	Nasal	1574.62 ± 177.06	3.34 ± 0.38
Free insulin, s.c.	0.25 IU/kg	Subcutaneous (s.c.)	2356.41 ± 106.19	100
Free insulin + PTD	1 IU/kg	Nasal	3643.49 ± 425.36	38.66 ± 4.51
3–3 formulation	1 IU/kg	Nasal	5722.18 ± 705.14	60.71 ± 7.48
3–5 formulation	1 IU/kg	Nasal	4322.47 ± 576.51	45.86 ± 6.12

AUC represents the area under the plasma insulin concentration-time curve from time 0 to 180 min.

The bioavailability (BA) was calculated using the following formula: BA% = (AUC_nasal_ × dose_s.c._)/(AUC_s.c._ × dose_nasal_) × 100%.

**Table 3.** Pharmacodynamics of nasally administered insulin formulations to alloxan-induced diabetic rats.

Formulation	Route	Insulin (IU/kg)	AAC0-240 min	Pharmacological availability (%)
Free insulin, nasal	Nasal	2	2156.65 ± 1070.68	4.01 ± 1.99
Free insulin, s.c.	S.c.	1	26897.33 ± 772.31	100
3–3 formulation	Nasal	2	26537.27 ± 1455.91	49.33 ± 2.71
3–5 formulation	Nasal	2	20181.39 ± 3035.38	37.52 ± 5.64

AAC represents the area above the blood glucose level-time curve from time 0 to 240 min.

The pharmacological availability (PA) was calculated using the following formula:

PA% = (AAC_nasal_ × Dose_s.c_)/(AAC_s.c_ × Dose_nasal_) × 100%.

### *In vivo* toxicity analysis of formulated insulin + PTDs

**Figure 3.**
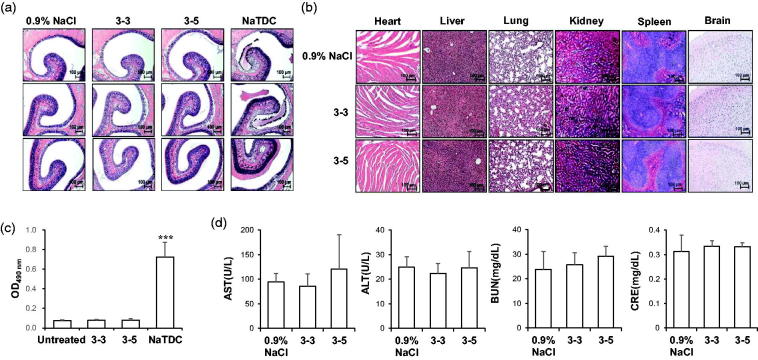
*In vivo* toxicity analysis of intranasal insulin + PTD formulations. (a–d) Normal mice were treated as indicated once a day for 10 d (*n* = 5). (a and b) Representative images of H&E staining of nasal cavity (a) and main organs (b). (c) OD_490nm_ means LDH activity from nasal wash solution. 5% NaTDC was used as positive control known to be toxic. ***p < .001 by Student’s *t*-test compared to untreated group. (d) Serum level of biochemical variables following nasal administration once a day for 10 d. Data are presented as mean ± s.d.

The use of intranasal delivery has been also limited by its toxicity to nasal membrane by enzyme inhibitors and nasal permeation enhancers (Fortuna et al., 2014). To evaluate the damage to nasal membrane, normal mice were intranasally administered insulin once in a day for 10 d, and sacrificed, and histological analysis of each nasal mucosa conducted after H&E staining (Figure 3(a)). While the NaTDC-treated group showed destruction of nasal mucosa, both 3–3 and 3–5 formulations showed histology similar with that of the 0.9% NaCl-treated group, indicating that these formulations did not induce topical toxicity. To evaluate the systemic toxicity in mice, other major organs were examined and no unusual pathological changes were observed in all experimental groups (Figure 3(b)). Moreover, low LDH activities of both formulations treated groups also confirmed minimal toxicity on nasal membrane (Figure 3(c)). Serum biochemical analysis was also performed from blood samples from the same experimental sets (Figure 3(d)). AST and ALT, indices of hepatocyte damage, were not altered, by formulated insulin + PTD. BUN and CRE, indicators for kidney damage, also did not change in the cases of 3–3 and 3–5 formulations, suggesting that mice maintain normal health during the intranasal administration. All these results confirm the safety of both formulations in mice, indicating their suitability for use in therapy.

### Stability analysis with *in vivo* study

**Figure 4.**
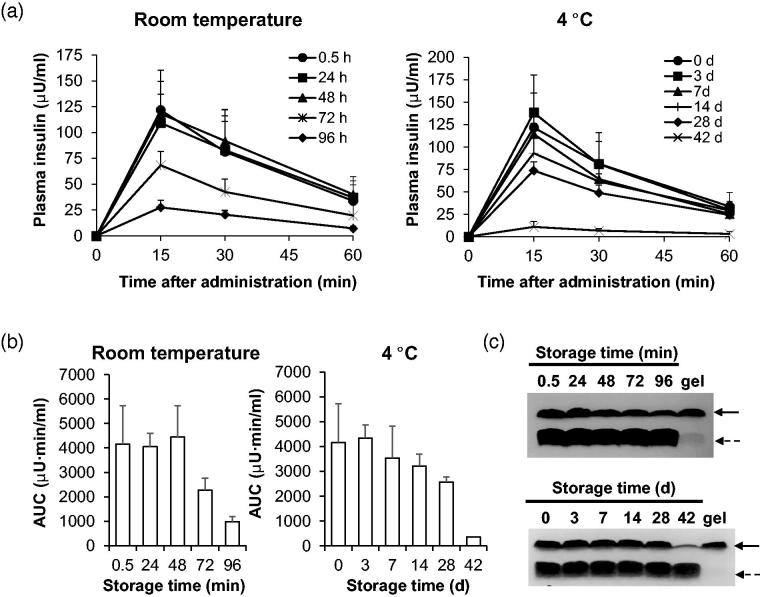
Biological evaluation of 3-3 formulation’s stability at two temperatures. (a, b) After incubation of indicated time at room temperature or 4 °C, insulin + PTD in 3–3 formulations was intranasally administered into normal rats. Dose of insulin administered was 1 IU/kg. (a) Blood was collected after various period time of administration, then plasma insulin level was measured by ELISA. (b) Areas under the curves were calculated for each group of rats. (c) Soluble insulin + PTD in 3–3 formulations was analyzed by Tricine-SDS PAGE under non-reducing conditions. Solid line arrow, insulin; Dashed line arrow, PTD. Data are presented as mean ± s.d.

We also assessed the stability of formulations and also requirements for their storage until use. We focused on stability studies of 3–3 formulation because the pharmacological and BA of insulin in alloxan-induced diabetic rats treated with 3–3 formulation were higher than those treated with 3–5 (Tables 2 and 3). Since the length and the temperature of storage are important factors that affect the viability of the formulation, we evaluated stability of insulin + PTD in 3–3 formulation at two temperatures: room temperature and at 4 °C. We intranasally administered normal rats with insulin + PTD in 3–3 formulation stored at the two conditions, and determined plasma insulin levels by ELISA and evaluated the levels of insulin delivered (Figure 4(a)). At both temperatures, prolonged storage resulted in decreased delivery of insulin in rats. Storage at room temperature maintained the levels of insulin delivered up to 48 h, while 4 °C-storage extended the period up to 7 d. As a pharmacokinetic parameter, area under curves (AUCs) for each group of rats were calculated (Figure 4(b)). Consistently, AUC value for the plasma insulin level-time graph remained similar up to 48 h at room temperature. However, 72- and 96-h storages decreased AUC value by 45% and 77%, respectively. When stored at 4 °C, the AUC value was maintained at more 85% level up to 7 d. But storage for more than 28 d showed drastic decrease. Thus, stable insulin delivery can be expected when insulin + PTD in 3–3 formulation is stored for 48 h at room temperature or for 7 d at 4 °C. We also confirmed time-dependent decrease in amount of insulin in 3–3 formulation with PTD by coomassie brilliant blue staining of supernatants from stored formulation (Figure 4(c)). Unlike insulin, the amounts of PTD did not change for up to 96 h at room temperature and 42 d at 4 °C. At the longest storage time at each temperature, we observed the formation of opaque gel on the surface of the container. To identify constituents of the gel, we performed the Coomassie brilliant blue staining on sonicated gel samples and found to be mostly insulin. This suggests that insulin aggregates on prolonged storage which results in reduced delivery of insulin. Based on these findings, insulin + PTD in 3–3 formulation should be stored at 4 °C rather than room temperature.

## Conclusions

In this study, we identified optimized formulation for insulin + PTD for enhanced intranasal delivery of insulin to rats. Two formulations (3–3 and 3–5 formulations) proved most suitable based on measurement of plasma insulin levels in normal rats based on pharmacokinetic, pharmacodynamic and toxicity studies. We also confirmed that both formulations improved delivery of insulin and blood glucose-lowering effects with minimal toxicity in diabetic rat models. We also established optimal storage conditions for the formulations. These findings should promote the nasal insulin delivery for the treatment of diabetes and also using TCTP-PTDs as a suitable carrier for delivery of a variety of other macromolecule.
